# The Effects of Combined Physical and Cognitive Interventions on Direct and Indirect Fall Outcomes for the Elderly with Mild Cognitive Impairment: A Systematic Review

**DOI:** 10.3390/healthcare10050862

**Published:** 2022-05-06

**Authors:** Hai Mai Ba, Jiyun Kim

**Affiliations:** 1 Faculty of Nursing, Hue University of Medicine and Pharmacy, Hue University, Hue 49000, Vietnam; mbhai@huemed-univ.edu.vn or; 2 School of Nursing, Gachon University, Incheon 21936, Korea

**Keywords:** cognitive therapy, elderly, falls, mild cognitive impairment, physical training

## Abstract

This review was intended to determine the effectiveness of physical and cognitive training (PCT) on falls and fall-related factors and cognitive function among community-dwelling elderly people with mild cognitive impairment (MCI). A systematic literature search was performed of the MEDLINE, CINAHL, Web of Sciences, Scopus, ProQuest, Embase, and Google Scholar databases for articles published from 2010 to 2020. The studies that combined PCT to assess their impacts on fall outcomes both directly and indirectly were included. Study quality was assessed using the standardized JBI Critical Appraisal Tool for RCTs. The standardized data extraction tool from JBI-MAStARI was used to extract data of included studies. Seven RCTs involving 740 participants were included. The overall fall incidence did not significantly decrease after the interventions. However, PCT significantly impacted the cognitive function and physical activities of elderly people with MCI, particularly improving their balancing ability, gait speed, muscular strength, and executive functions. This study indicated that combining PCT improves balance ability, gait speed, and executive functioning in the elderly with MCI, which may help to minimize fall occurrence.

## 1. Introduction

The elderly with cognitive impairment are prone to falls [1,2], and the relationship between fall frequency and cognitive function has been identified in several studies [3,4,5,6]. In particular, the fall frequency is higher in the elderly with mild cognitive impairment (MCI) than those without MCI [7], which is related to a decline in their motor function, such as the manifestation of a slow gait [8]. 

Since elderly people with cognitive impairment are often excluded from general fall-prevention programs for the elderly in local communities [9], a fall-prevention program needs be developed for this population. Cognitive interventions, including memory, attention, and computer-guided training, as well as behavioral therapy [10], have been indicated in systematic reviews to improve fall-associated factors such as gait speed, balance, and functional mobility when used as part of a fall-prevention program [11,12]. The pathophysiological mechanism underlying how cognitive training (CT) is effective in preventing falls is currently unclear, but exercise therapy combined with CT is necessary for preventing falls among elderly people [13]. Two previous studies reviewed CT and physical training (PT) programs for the elderly with cognitive impairment [11,12]: one reviewed physical and cognitive interventions for the elderly with cognitive impairment either by diagnosing or assessing their overall cognition [11], and the other reviewed exercise and CT for elderly people with MCI [12]. At the time of writing, no other systematic review has been conducted to investigate the combined PT and CT intervention method in only elderly people with MCI in terms of fall outcomes. Therefore, the present study will contribute to update the evidence from the studies using only a combination of physical and cognitive training (PCT) for the elderly with MCI. 

The primary goal of this study was to summarize the current evidence of RCTs on the efficacy of combining physical and cognitive interventions in preventing falls and fall risk factors, such as balance, muscular strength, gait, and cognitive function. The finding of this review may help to address optimal intervention for fall prevention in community-dwelling elderly with MCI. 

To comprehensively and unbiasedly synthesize the effects of PCT on fall prevention in the elderly with MCI, we used guidelines for confirming the quality of a study for systematic reviews of randomized controlled trials (RCTs). Previous studies have utilized these guidelines, and both above-mentioned studies used the 10-item Joanna Briggs Institute Reviewers’ Manual instrument to select the studies [14]. In the present study, we selected studies for review using 13 criteria according to the new JBI guidelines [15]. 

## 2. Methods

### 2.1. Inclusion Criteria

Studies were selected based on the PICO model [16]: (1) Target participants were people older than 60 years who were diagnosed or identified as having an MCI based on screening tools or physician diagnoses; and (2) type of interventions was described as combined PCT programs. Physical training includes exercise or physical activities aimed at improving balance, muscular strength, gait, or daily living activities. CT programs were included that aimed to improve the global cognitive function, memory, and scores on executive function subscales. The delivery method of the interventions in relevant studies did not limit their inclusion in this review, such as group interventions or having both intervention and control groups (with a 1:1 ratio). Examples of interventions were dual-task exercise [17,18], which combined simple number calculations and memory games given during aerobic exercise, and sensor-based balance training with motion feedback [19]); (3) the comparison group could be a control group related to no training, health promotion, routine medical care, only physical interventions, only CT programs, or a waitlisted group; (4) falls can be measured directly or indirectly. Direct measures include the number of fallers or the incidence. Indirect and substitute methods consider the physical and cognitive factors associated with falls. Physical factors, such as balance, muscle force, and gait, and cognitive factors, such as cognition, memory, and executive function, were taken into consideration.

### 2.2. Search Strategy

This review only included randomized controlled clinical trials and experimental studies that had been reported on in English. The MEDLINE, CINAHL, Web of Science, Embase, Scopus, Google Scholar, and ProQuest databases were searched for relevant articles. The following Boolean search string was used for the search: (“cognitive training” OR “cognitive intervention” OR “brain training” OR “cognitive exercise” OR “memory training”) AND (“physical exercise” OR “combined” OR “cognitive-motor” OR “dual-task training”) AND (“aged” OR “older people” OR “elderly”) AND (“MCI”). The full texts of articles included in this study needed to be available for evaluation and published between January 2010 and December 2020. Non-original research, protocol studies, and intervention development studies were excluded.

### 2.3. Study Selection and Quality

One investigator performed the review independently (H.M.B.). It primarily consisted of reading over the titles and abstracts of all studies found through database searches. Second, according to the inclusion or exclusion criteria, two investigators (H.M.B. and J.K.) independently evaluated the obtained full-text studies. Studies that did not meet the inclusion criteria were excluded, and selected studies were agreed upon by two investigators and evaluated for quality in the following phase. Study quality was assessed using the standardized JBI Critical Appraisal Tool for RCTs [15] This is the 13-item checklist that covers quality issues concerning randomization, concealed allocation, the groups’ similarities at baseline, the blinding type, attrition, treatment protocol, follow-up, use of outcome measures, statistical analysis, and RCT type. Each article was independently scored by the two investigators as yes, no, unclear, or not-applicable responses for each item. Any difference in the score of each item was subsequently discussed and resolved by the two investigators. Prior to critically reviewing the studies, the investigators discussed scoring criteria and agreed to only include studies with at least seven “yes” responses.

### 2.4. Data Extraction and Synthesis

Quantitative data were extracted from the articles included in the review using the standardized data extraction tool from JBI-MAStARI. The extracted data contained detailed information on the population’s characteristics, intervention features, outcomes, and measures (Table 1). Due to the variability in the outcome measures of the included studies, the findings on fall outcomes were then synthesized and presented in narrative form, and meta-analysis was not performed.

## 3. Results

**Figure 1:**
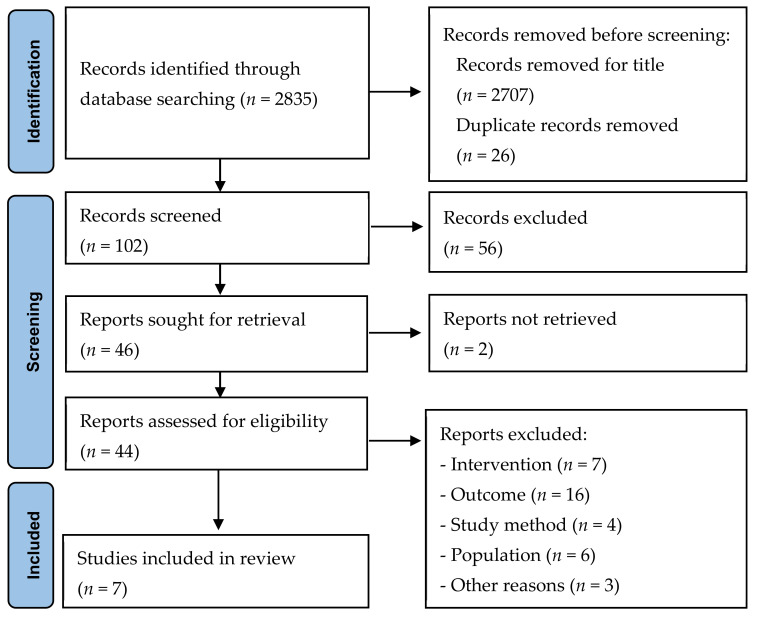
Flow diagram of study retrieval and selection.

The search process identified 2835 published and unpublished studies for potential inclusion in the review. Screening the publication titles resulted in 2707 articles being excluded. The remaining 128 articles were then consolidated, with 26 duplicates removed and 56 articles excluded after abstract screening. The full manuscripts of the remaining 46 articles were obtained for full-text reading, which resulted in 38 articles being excluded for various reasons as listed in Figure 1 (see Supplementary Table S1). Eight articles were obtained for critical appraisal, with one being excluded due to the methodological assessment score being under 7, leaving seven being finally included in the review. 

### 3.1. Characteristics of Included Studies

The total number of participants in all the included studies was 740. The mean age of these participants ranged from 69.5–79.2 years. All participants had MCI identified via diagnosis or cognitive screening test results. The seven included studies comprised two conducted in the United States, and the remaining ones were conducted in France, South Korea, Japan, Taiwan, and the Philippines. Table 1 lists the study characteristics of the seven articles on the effects of PCT on preventing falls and the risk of falls, including cognitive and physical outcomes. Only two of the included studies measured direct outcomes of fall incidence [17,20], with the other five studies measuring indirect outcomes of falls related to cognitive and physical risk factors [18,19,21,22,23]. Three RCTs focused on individual-based computerized or virtual-reality-based intervention strategies [21,22,23], and four used group-based strategies of PCT [17,18,19,22].

### 3.2. Methodological Quality

Table 2 lists the quality assessment scores of all included studies. All studies satisfied the method of true randomization, similar groups at baseline, consistently measured outcomes, and trial design appropriation. Allocation concealment was unclear in three studies [18,22,24]. Regarding the criteria for assessing blinding methods, one study blinded participants to their treatment assignment [20], and four studies blinded the outcome assessor [17,20,21,24]. However, one of the studies blinded those delivering the intervention to the treatment group. In four studies, the assignment groups were exposed to other activities that may have affected study outcomes, or it was unclear whether this had occurred [18,19,21,24], and four studies did not use intention-to-treat analysis [19,21,22,24]. One study used many statistical tests and had a small sample; its *p*-values could not be interpreted as estimates of type I and type II error probabilities [21]. Finally, one study was excluded since it had a methodological quality appraisal tool score lower than 7 [24].

### 3.3. Effectiveness of PCT in Preventing Falls and Risks of Falls in the Elderly with MCI

The articles in this review measured falls either directly or indirectly. Direct measures included the number of falls, number of fallers, or fall rate, and indirect outcomes included physical and cognitive risk factors (Table 3).

#### 3.3.1. Direct Fall Outcomes

Overall, the number of falls did not decrease significantly after the intervention. However, the fall outcomes were only measured in two studies: in one as a major outcome [20] and the other as a minor outcome [17]. The periods of measuring fall outcomes also differed. Lipardo and Tsang (2020) found no significant difference in the fall incidence rate between times and groups after 12 weeks (*p* = 0.152) and 36 weeks (*p* = 0.954). Shimada et al. (2018) reported that falls in daily life did not differ significantly between the intervention and control groups after 40 weeks (8.4% and 9.2%, respectively, *p* > 0.05).

#### 3.3.2. Indirect Fall Outcomes/Risks of Fall

Physical outcomes: three outcomes were repeatedly reported to be associated with fall risk across the included studies: balance, gait, and muscular strength. 

Overall, after the interventions, dynamic balance and functional mobility improved and positively changed in the combined-intervention group. The timed-up-and-go (TUG) test [18,19,20] and get-up-and-go (GUG) test were commonly used to assess functional mobility and balancing ability [22]. These four studies found significant improvements in balance and physical activity according to follow-up times and in comparison groups as follows: (1) compared with the control group, TUG test score in the experimental group improved after 24 weeks (OR = −0.8, 95% CI = −0.4 to −1.4, *p* < 0.01) [18]; (2) the PCT and CT groups exhibited better functional ability compared with the waitlist group at week 36, and the PCT group also significantly improved at weeks 12 and 36 [20]; (3) the TUG test score in the PCT group decreased significantly after 12 weeks and 6 months both compared with the control group and within the group [19]; and (4) physical ability measured using the GUG test in the exer-tour group increased significantly more than in the exer-score group (*p* = 0.001) [22].

Gait performance was measured in four studies, of which two measured it in three conditions: (1) single-task walking, (2) complex walking, and (3) dual-task walking. The findings indicated that complex walking capacities had a larger range of improvement in the PCT group than in the groups in the first study that engaged in a single training [19]. In the second study, both single-task and motor dual-task gait performance improved significantly after 12 weeks of training. However, only the virtual reality (VR) group improved in cognitive dual-task gait performance and cadence dual-task cost (DTC). Furthermore, the VR group outperformed the PCT group in cadence DTC (*p* < 0.05) [21]. In the other studies, gait performance was assessed using the 10-meter walk test, habit walking, and fast walking. The PCT group did not improve significantly compared with the control group following the intervention [20,23].

To evaluate lower limb muscular strength, sit-to-stand time, and a 30-second chair-stand test was used to measure the effectiveness of the combined interventions in two studies. Only one found that the sit-to-stand time improved significantly compared with the control group following 24 weeks of intervention [18]. The other study suggested that there was no significant change in lower limb muscular strength in the 30-second chair-stand test after 12 and 36 weeks. Only the physical exercise group showed a statistically significant increase in lower limb muscular strength between the baseline and at 12 and 36 weeks (*p* < 0.05) [20].

The findings of our included studies also indicated that daily step counts and physical activity intensity in the combined intervention group improved compared with the control group during the study periods of 24 weeks (*p* < 0.01) [18] and 40 weeks (*p* < 0.001) [17].

#### 3.3.3. Cognitive Outcomes

Global cognitive functions were mostly measured using the Mini Mental State Examination (MMSE), Montreal Cognitive Assessment (MoCA), and modified Alzheimer’s Disease Assessment Scale-Cognitive Subscale (ADAS-Cog). Overall, the combined interventions exerted mixed effects on general cognitive functions. The combined intervention group showed significantly greater differences between scores on the MMSE [17] and ADAS-Cog [18] compared with the control group after the intervention. In the two other studies, the MMSE [18] and MoCA [23] scores did not change after the intervention. 

Numerous neuropsychological tests were used to evaluate the executive functions in different cognitive function domains, including the Stroop test, symbol-digit substitution test, the color trails test, the digit span test (DST), the trail-making test (TMT), Rey’s auditory verbal learning test, and the verbal fluency test. Of these, the TMT was commonly used to evaluate attention domain outcomes in four studies [17,18,21,23], two of which found significant improvements in attention ability in the PCT group after the intervention [17,21]. The DST was measured in three studies, two of which found a significant improvement in working memory ability. Park et al. (2019) found significantly greater changes in working memory in the combined intervention than in the control group (*p* < 0.05) after 24 weeks of intervention. Donnezan et al. (2018) also reported improvements in attention capacity and working memory for all groups except the control after 12 weeks, and working memory was still improved relative to the baseline after 6 months for the PT and PCT participants. The Stroop test was used in four out of seven studies that evaluated inhibitory control. This assesses the ability of a subject to inhibit an automatic behavior (reading a word) and perform a controlled behavior (saying the front color of the word). Overall, the PCT improved the ability to inhibit executive functions at 12 weeks to 6 months after the intervention. Anderson-Hanley et al. (2018) found that the Stroop test score improved significantly in both the exer-tour (t (1, 5) = −2.6, *p* = 0.049) and exer-score (t (1, 6) = −5.5, *p* = 0.001) groups over 6 months. However, after 3 months, the exer-tour group experienced a significant and moderate effect, while the exer-score group experienced the little effect, as did a game-only condition. Liao et al. (2019) found that both the VR and PCT groups exhibited significant improvements in Stroop test scores, but there was no significant difference between the groups. Donnezan et al. (2018) indicated an improvement in Stroop test scores after combined PCT, but the difference was not statistically significant (*p* = 0.06).

## 4. Discussion

This study is the systematic review that combined PCT intervention for preventing falls and identified the effects. Seven studies were selected for review when using the JBI tool to assess the quality of included studies [15]. The objectives of this systematic review were to determine what kinds of combination of cognitive and physical training have been implemented according to their effects on cognitive function and fall rate and fall-related factors. 

A PCT intervention was found to be useful for improving cognitive and physical function in the elderly with MCI [25] and seemed to prevent falls by improving balance ability [11,12]. In the present study, cognitive and physical function training programs involved dual-task training [17,18,21], and interventions were developed that could immediately determine subject responses by using on-screen instructions for an ankle-reaching exercise [23], using a bicycle with a video screen [19,22], or VR [21]. Cognizing was performed during physical activity, and the subjects participated in word games [17,18] or naming flowers or animals [21].

A training program developed using VR was found to improve the cognitive and physical functions of the elderly with MCI [26]. In another systematic review study, physical activity combined with VR was associated with improved cognitive function among the elderly either with or without MCI [27]. On the other hand, VR training helped to improve the overall cognitive function of the elderly with MCI but had no significant effect on memory [28]. Most of the included studies found strengthened evidence of a significant difference by comparing a VR-combined training program with a control group. In the study of Liao et al. (2019), the PCT program combined with VR induced improvements in some of the TMT scores and some of the gait measures compared with the traditional intervention program, but its other outcomes did not differ significantly between the VR and traditional intervention groups [21]. Further evidence needs to be obtained by comparing the effectiveness of cognitive and physical training programs combined with VR and traditional programs. However, since a VR program has the advantage of providing a controlled and safe environment for cognitive rehabilitation and is easy to reproduce in daily life, it is a useful tool that is applicable to the elderly with MCI [2].

Two of the reviewed studies directly measured the frequency of fall occurrence [17,20], but the small number of reviewed studies made it difficult to conclude that the combined program reduced the frequency of fall occurrence. Moreover, since the dual-task intervention [17] and PCT [20], which directly measured the frequency of falls as the intervention outcome, did not reveal significantly different results between groups, it was difficult to conclude that it helped to prevent falls. 

Instead of directly measuring fall incidence, many studies measured variables such as balance, mobility, and gait related to falls, which were also used in many studies with interventions aimed at enhancing physical activity to prevent falls. Various types of intervention also induced significant changes in fall-related variables. For balance measurements, sway was measured using a wearable sensor. In one included study, the intervention group showed better sway results than the control group [23]. Measuring balance using the TUG test indicated that the intervention group had improved significantly compared with the control group in two studies [19,20]. There were also studies that determined the effect of combined PCT by measuring the mobility improvements of the elderly with MCI. Moderate-to-vigorous intensity physical activities were commonly measured [17,18], and the number of daily steps was also measured [17]. Gait was also mostly measured as a fall-related variable, and the effect of the PCT in improving gait performance was indicated, which was measured using the gait speed [18,19] and the dual-task gait [21]. Fear of falling was also a variable used to measure the effectiveness of a program, and there was a significant difference between the intervention and control groups [23]. However, it was difficult to calculate the effect size since the tools used to measure the effectiveness of fall prevention were too heterogeneous, but the PCT was still considered to help prevent falls in the elderly with MCI. 

The tools used frequently to measure differences in cognitive function before and after a program were the Stroop test [19,21,22], MMSE [18], and TMT [17,21]. Overall, the findings of the present study indicated improvements in the attention, working memory, and inhibition domains of executive function in the PCT group in most of the included studies. This is comparable to the previous systematic review by Law et al. (2014) indicating that three out of five studies found significant increases in global cognitive function, memory, executive function, or attention [29]. Gavelin et al. (2021) also found similar results, with PCT significantly improving cognitive outcomes, including executive function, short-term and working memory, processing speed, and global cognition, but not visual processing [25]. However, these effects were inconsistent; the systematic review of Karssemeijer et al. (2017) indicated that the cognitive domains of executive function/attention and memory did not significantly affect the combined intervention group [30]. Together, these findings indicate that further research is needed to validate the efficacy of the combined intervention on cognitive outcomes, and when performing a physical activity intervention program combined with CT in the future, it is recommended to compare with previous studies using the same tools. 

The continuation of each intervention activity is important for preventing falls in the elderly with cognitive impairment. In this regard, it is also important to determine whether an intervention activity is easy to perform in daily life so that the subject can practice it over the long term. It is therefore important to develop a program that can be easily performed by the elderly in the community and that can be continued after the intervention concludes. Only two studies followed up after the intervention period [19,20], but it was not confirmed whether the subjects continued training during that period.

The limitations of this study were as follows: The number of reviewed studies was small due to combining PT and CT and selecting interventions targeting MCI. This approach made it difficult to pool the study results to carry out a meta-analysis. In addition, the effect size could not be measured since the outcomes in the reviewed studies were too heterogeneous. This was because the measurement variables related to falls and the variables measuring cognitive function were both diverse. However, this study has implications that can be referred to when planning a program to prevent falls among the community-dwelling elderly with MCI. 

## 5. Conclusions

This was systematic review studies including to directly and indirectly observe the effects of PCT on fall outcomes in community-dwelling elderly with MCI. Although the combined intervention did not induce a direct decrease in fall rates, it did exert a strong indirect effect on reducing fall risk factors for the elderly with MCI, including their balance, gait speed, muscular strength, and cognitive function. However, we still cannot infer that the combined intervention reduced the fall incidence compared with the single intervention because only two of the included studies reported fall rates in their respective trials. Therefore, additional studies on the direct effect of this intervention on fall rates in elderly people with MCI are needed. Our findings also add to the existing understanding of the impact of PCT on most physical and cognitive domains. Finally, it is advised that when implementing a physical activity intervention program combined with CT in the future, the same tools as those used in previous research should be used in the analysis. 

## Figures and Tables

**Table 1:** Descriptive summary of fall-prevention intervention studies on the elderly with mild cognitive impairment (MCI) (*n* = 7).

Study	Setting	Participants	Outcomes of Interest	Length of Intervention	Intervention Setting	Intervention
Lipardo et al. (2020) [20]	Philippines: Community-based program	• Gr1: *n* = 23 • Gr2: *n* = 23 • Gr3: *n* = 23 • Gr4: *n* = 23 Age 69.5 y, MMSE score N/A, MoCA score 18.3 (4.6)	• Fall outcomes: (1) Information on fall incidence • Risk of falls: (1) Physiological profile assessment—short form • Dynamic balance and functional mobility: (1) TUG test • Walking speed: (1) The 10-meter walk test • Lower extremity muscular strength: (1) 30-second chair-stand test	12 weeks	Group	Gr1: PT • Duration: 60–90 min/session; 3 times/week; 12 weeks Gr2: PT and PCT • Duration: 60–90 min/session; 3 times/week; 12 weeks Gr3: CT • Duration: 60–90 min/session; 1 time/week; 12 weeks Gr4: Usual daily routine
Liao et al. (2019) [21]	Taiwan: Community	• Gr1: *n* = 21, age 75.5 (5.2) y, MMSE score 27.2 (1.9) • Gr2: *n* = 21, age 73.1 (6.8) y, MMSE score 27.2 (1.6)	• Executive function: (1) TMT (2) SCWT • Gait performance: (1) Single-task gait (2) Cognitive dual-task gait (3) Motor dual-task gait	12 weeks	Group	Gr1: VR-based PCT. Gr2: PCT • Duration: 60 min/session, 3 times/week, 36 sessions over 12 weeks
Park (2019) [18]	South Korea: Community	• IG: *n* = 25, age 70.6 (6.46) y, MMSE score 24.6 (2.6) • CG: *n* = 24, age 72.8 (5.37), MMSE score 24.4 (3.1)	Primary outcomes: (1) K-MMSE, (2) ADAS-Cog Secondary outcome: • Cognitive assessment: (1) DST, (2) TMT, (3) SDST. • Physical function assessment: (1) Grip strength, (2) TUG, (3) sit-to-stand time • Physical activity measurements (1) MVPA, (2) step count	24 weeks	Group 1:1	IG: Dual-task exercise program. • Duration: 110 min sessions/week over 24 weeks CG: No intervention
Donnezan et al. (2018) [19]	France: Urban elderly clubs	• Gr1: *n* = 21, age 77.1 (1.44) y, MMSE score 28.2 (0.43) • Gr2: *n* = 19, age 76.3 (1.5) y, MMSE score 27.3 (0.42). • Gr3: *n* = 21, age 75.2 (1.3) y, MMSE score 28.1 (0.36) • Gr4: *n* = 15, age 79.2 (4.0) y, MMSE score 27.3 (0.5)	• Executive measures: (1) Matrix Reasoning Test, (2) SCWT, (3) DSF, (4) DSB • Motor measures: (1) Rockport test (VO_2_max), (2) TUG test, (3) single-task walking, (4) complex walking (gait speed WSC test: bw, w, c), (5) dual-task walking	12 weeks	Group	• Gr1: PT • Gr2: CT • Gr3: PCT • Duration: two 1-hour sessions/week over 12 weeks; same to all groups. Gr4: No intervention (usual lifestyle)
Shimada et al. (2018) [17]	Japan: Residential suburb of Nagoya	• IG: *n* = 154, age 71.6 (5.0) y, MMSE score 26.6 (1.8) • CG: *n* = 154, age 71.6 (4.9) y, MMSE score 26.8 (1.8)	• Functional outcomes: (1) MMSE, (2) WMS-LM II, (3) RAVLT • Cognitive outcomes: (1) VFT—letters, (2) VFT—category, (3) TMT • Mobility: (1) Total daily steps, (2) MVPA	40 weeks	Group 1:1	IG: Combined activity program: physical and cognitive activities. • Duration: 90 min/ sessions/ 40 weeks CG: Health education • 90 min health promotion classes thrice during the 40-weeks trial period
Anderson-Hanley et al. (2018) [22]	USA: Community	• Gr1: *n* = 46 • Gr2: *n* = 45 • Gr3: *n* = 20 Age 78.1 (9.9) y, MMSE score N/A, MoCA score 23.7 (3.1) (screened as MCI” based on MoCA score < 26)	Primary measures: • Executive function: (1) Stroop test, (2) color-trails test, (3) DST Secondary measures: (1) Ecological validity, (2) verbal memory immediate and delayed recall, (3) get-up-and-go test	6 months	Group	• Gr1: exer-tour: physical exercise interactive with relatively passive, low cognitive load, virtual scenic bike tour. • Gr2: exer-score: interactive physical exercise a relatively high effort, high cognitive demand, video game • Gr3: game-only: the same videogame operated by a joystick or keyboard • Duration: 20 min/twice/week and increase 45 min/3–5 times/week for 6 months.
Schwenk et al.(2016) [23]	USA: Cleo Roberts Memory and Movement Disorders Center	• IG: *n* = 12, MoCA score 23.3 (3.1) • CG: *n* = 10, MoCA score 22.4 (3.0). Age 78.2 (8.7) y.	Balance (EC, EO): (1) CoM sway, (2) ML CoM sway, (3) AP CoM sway Gait: (1) Habitual walking, (2) fast walking Fear of falling: (1) FES-I Cognitive performance: (1) MoCA, (2) TMT	4 weeks	Group 1:1	IG: Sensor-based balance training with motion feedback • Duration: 45 min/session; 2 training sessions/ week for 4 weeks. CG: No training.

IG, intervention group; CG, control group; Gr, group; MMSE, Mini Mental State Examination; MoCa, Montreal Cognitive Assessment; Korean version of the KMMSE; TUG, timed-up-and-go; VFT, verbal fluency test; DST, digit span test; TMT, trail-making test; TMT-B, the Chinese version of TMT; MVPA, moderate-to-vigorous intensity physical activity; WMS-LM II, Wechsler Memory Scale Revised Logical Memory II; RAVLT, Rey auditory verbal learning test; ADAS-Cog, modified Alzheimer’s Disease Assessment Scale Cognitive Subscale (ADAS-Cog); SDST, symbol–digit substitution test; SCWT, Stroop color and word test; StroopA/C, ratio of the executive function component of the task; DSF, digit span forward test; DSB, digit span backward test; FES-I, short version of the Falls Efficacy Scale International; DRT-II, disjunctive reaction time; NHPT, Nine Hole Peg Test; WSC: Walking Stroop Carpet test; bw, black and white; w, word; c, color; DTC, dual-task cost; PT, physical training; CT, cognitive training; PCT, physical and cognitive training; VR, virtual reality; EC, eyes closed; EO, eyes open; CoM, center of mass; ML, medial–lateral; AP, anterior–posterior.

**Table 2:** Methodological assessment scores of included studies on the JBI Critical Appraisal Tool.

Study	Q1	Q2	Q3	Q4	Q5	Q6	Q7	Q8	Q9	Q10	Q11	Q12	Q13	Total Score
Park et al. (2019) [18]	Y	U	Y	N	N	U	N	Y	Y	Y	Y	Y	Y	8
Shimada et al. (2018) [17]	Y	Y	Y	N	N	Y	Y	Y	Y	Y	Y	Y	Y	11
Lipardo et al. (2020) [20]	Y	Y	Y	Y	U	Y	Y	Y	Y	Y	Y	Y	Y	12
Donnezan et al. (2018) [19]	Y	Y	Y	N	N	N	N	Y	N	Y	Y	Y	Y	8
Liao et al. (2019) [21]	Y	Y	Y	N	N	Y	N	Y	U	Y	Y	N	Y	8
Anderson-Hanley et al. (2018) [22]	Y	U	Y	N	N	N	Y	Y	N	Y	Y	Y	Y	8
Schwenk et al. (2016) [23]	Y	Y	Y	N	N	N	Y	Y	Y	Y	Y	Y	Y	10
Delbroek et al. (2017) [24]	Y	U	Y	N	N	Y	N	N	N	N	Y	Y	Y	6
Total (%)	100	70	100	30	0	60	40	90	60	90	100	90	100	Mean score: 8.87

Q1, random assignment; Q2, concealed allocation; Q3, baseline similar; Q4, blinding to allocations; Q5, blinding to treatment; Q6, blinding to assessor; Q7, identical treatment of groups; Q8, follow up complete; Q9, intention-to-treat; Q10, outcomes measured in the same way; Q11, reliable measurement of outcomes; Q12, appropriate statistical analysis; Q13, appropriate trial design; N, no; Y, yes; U, unknown.

**Table 3:** Falls and falls-related outcome results and findings of statistical significance.

Study	Measurement	Results	Significant Difference between Groups	Effective
FALLS OUTCOME				
Lipardo et al. (2020) [17]	Fall incidence rate in: previous 12 mo; post intervention (12 and 36 weeks)	Gr1: pre. *n* = 6; post. *n* = 0 and 4 Gr2: pre. *n* = 6; post. *n* = 5 and 6 Gr3: pre. *n* = 7; post. *n* = 3 and 5 Gr4: pre. *n* = 6; post. *n* = 5 and 5	At 12 weeks (*p* = 0.152) or 36 weeks (*p* = 0.954), there were no significant differences in fall incidence rates according to time or group.	No
Shimada et al. (2018) [18]	Total number of falls in: post intervention (40 weeks)	IG: post. *n* = 11 CG: post. *n* = 13	Fall in daily life was not significant different between-group (*p* = 0.811)	No
FALLS-RELATED OUTCOMES				
	Balance ability: Timed-up-and-go test (TUG); get-up-and-go test (GUG)			
Lipardo et al. (2020) [20]	Mean TUG (s) at: baseline; 12th week, 36th week follow up	Gr1: 10.7 (2.8); 9.34 (2.0); 9.0 (1.3) Gr2: 10.6 (3.0); 10.45 (2.6); 9.6 (1.4) Gr3: 9.1 (3.0); 8.91 (2.6); 8.6 (2.0) Gr4: 10.6 (4.0); 11.2 (3.5); 11.1 (2.6)	Significant improvement in dynamic balance based on timed-up-and-go test in the combined physical and cognitive training group (9.0 s with *p* = 0.001) and in the cognitive training alone group (8.6 s with *p* = 0.012) compared to waitlist group (11.1 s) at 36 weeks.	Yes
Park (2019) [18]	Mean TUG at: baseline; 24 weeks follow up	IG: 10.1 (3.1); 8.9 (3.4) CG: 9.7 (4.1); 9.5 (3.9)	Compared with the control group, timed-up-and-go test showed significant improvement after the intervention (*p* < 0.01)	Yes
Donnezan et al. (2018) [19]	Mean TUG at: baseline; 6 mo follow up	Gr1: 9.96 (1.75); 8.90 (1.21) Gr2: 10.24 (2.68); 9.97 (3.44) Gr3: 12.79 (2.17); 9.84 (1.18) Gr4: 11.65 (2.04); 1.58 (2.18)	The TUG test score in the PCT group decreased significantly after 12 weeks and 6 months both compared with the control group and within the group.	Yes
Anderson-Hanley et al. (2018) [22]	Mean GUG at: baseline; 6 mo follow up.	Gr1: 12.7 (2.2); 14.0 (1.5) Gr2: 10.3 (1.7); 11.0 (2.0)	The GUG test in the exer-tour group increased significantly more than in the exer-score group (*p* = 0.001)	Yes
	Gait performance			
Lipardo et al. (2020) [20]	Mean 10-meter walk test (m/s) at: baseline; 12th week and 36th week.	Gr1: Preferred speed: 1.08 (0.17); 1.11 (0.18); 1.13 (0.16) Fastest speed: 1.38 (0.23); 1.41 (0.25); 1.42 (0.24) Gr2: Preferred speed: 0.99 (0.18); 1.09 (0.18); 1.09 (0.18). Fastest speed: 1.24 (0.23); 1.39 (0.24); 1.38 (0.23). Gr3: Preferred speed: 1.13 (0.20); 1.20 (0.24); 1.12 (0.21) Fastest speed: 1.51 (0.30); 1.54 (0.31); 1.47 (0.29). Gr4: Preferred speed: 1.02 (0.23); 1.02 (0.21); 1.01 (0.17). Fastest speed: 1.32 (0.32); 1.33 (0.30); 1.31 (0.28).	The PCT group did not improve significantly compared with the control group following the intervention	No
Liao et al. (2019) [21]	Gait speed (cm/s) in single-task gait, cognitive dual-task gait, and motor dual-task gait at: baseline; 12-week follow up	Gr1: (VR group) • Single-task walking: 82.3 (29.1); 92.9 (28.5) • Complex walking: 68.1 (26.9); 82.5 (30.6) • Dual-task walking: 79.9 (29.9); 92.3 (32.8) Gr2: (CPC group) • Single-task walking: 89.3 (23.3); 100.19 (25.7) • Complex walking: 72.8 (25.9); 78.1 (33.2) • Dual-task walking: 86.5 (25.0); 96.1 (27.3)	Gait speed in three conditions significantly improved within VR group and CPC group except for cognitive dual tasks in CPC group. This gait speed did not significantly change between groups.	Yes within group No between groups
Donnezan et al. (2018) [19]	Gait speed in single task (cm/s) at: baseline; 6 month follow up	Gr1: 115.93 (18.6); 119.42 (17.89) Gr2: 111.34 (19.91); 112.58 (26.13) Gr3: 102.43 (12.60); 114.44 (16.03) Gr4: 99.64 (14.29); 90.56 (15.23)	Gait speed in the PCT group significantly improved after 6 months of intervention (*p* = 0.001) but did not change between groups.	No
Schwenk et al.(2016) [23]	Gait speed (m/s) in habitual walking and fast walking	IG: • Habitual walking: 0.98 (0.22); 1.05 (0.22) • Fast walking: 1.39 (0.35); 1.43 (0.34) CG: • Habitual walking: 1.06 (0.17); 1.10 (0.20) • Fast walking: 1.44 (0.22); 1.34 (0.37)	Gait speeds were nonsignificant between groups (*p* > 0.05)	No
	Muscular strength			
Park (2019) [18]	Mean sit-to-stand time: at baseline; 24 weeks follow up	Gr1: 18.0 (5.2); 16.9 (4.5) Gr2: 17.3 (4.7); 17.7 (4.8)	Compared with the control group, the sit-to-stand time showed significant improvement after the intervention (*p* < 0.01)	Yes
Lipardo et al. (2020) [20]	Median 30-second chair-stand test: at baseline, 12th week, 36th week	Gr1: 13 (3); 13 (2); 14 (2) Gr2: 13 (3); 15 (4.5); 15 (3) Gr3: 15 (5.5); 15 (5.5); 16 (5) Gr4: 14 (6); 15 (5); 13 (4)	No significant group effect was observed in the 30-second chair-stand test for lower limb muscle strength at 12 weeks (*p* = 0.186) and at 36 weeks (*p* = 0.110), but there was a significant time effect (*p* < 0.001)	No
	Global cognitive functions			
Shimada et al. (2018) [17]	Mean difference MMSE after 40 weeks follow up (OR, 95%CI)	IG: 0.0 (−0.4 to 0.4) CG: −0.8 (−1.2 to −0.4)	Compared with the controls, the combined activity group exhibited significantly greater score changes on the MMSE (difference = 0.8, *p* = 0.012)	Yes
Park (2019) [18]	Mean MMSE at: baseline, 24th week follow up Mean Modified ADAS-Cog at: baseline; 24th week follow up	IG: 24.6 (2.6); 24.8 (3.7) CG: 24.4 (3.1); 24.2 (3.0) IG: 26.2 (2.9); 24.6 (3.3) CG: 25.7 (3.1); 26.1 (2.7)	The combined intervention group showed significantly greater differences between scores on the ADAS-Cog (*p* < 0.01) compared with the control group after the intervention but did not change on the MMSE score (*p* = 0.06)	Yes for the ADAS-Cog test No for MMSE test.
Schwenk et al.(2016) [23]	Mean MOCA score at: baseline; 4th week follow up	IG: 23.3 (3.1); 23.7 (3.9) CG: 22.4 (3.0); 25.3 (1.9)	The MoCA scores did not change after the intervention.	No
	Executive functions:			
	The trail making test (TMT)			
Shimada et al. (2018) [17]	Mean difference TMT at: baseline; 40th week follow up (OR, 95%CI)	IG: −0.3 (−0.7 to 0.1) CG: 0.0 (−0.2 to 0.3)	The control and combined activity groups did not significantly differ in TMT score after intervention (*p* = 0.35)	No
Park (2019) [18]	Mean TMT-A at: baseline; 24th week follow up	Gr1: 25.3 (7.1); 23.1 (6.3) Gr2: 24.7 (6.2); 24.1 (6.7)	There were no significant differences in TMT-A between groups after 24 weeks (*p* = 0.1)	No
Liao et al. (2019) [21]	Mean TMT-B at: baseline; 12th week follow up.	Gr1: 179.22 (58.06); 134.21 (48.23) Gr2: 154.50 (63.50); 136.37 (48.58)	There were significant differences in TMT-B between groups after 12 weeks of intervention (*p* = 0.032)	Yes
Schwenk et al.(2016) [23]	Mean TMT-A, TMT-B at: baseline; 4th week follow up	IG: TMT-A: 51.8 (24.3); 46.0 (14.1) TMT-B: 149.2 (89.5); 155.6 (101.3) CG: TMT-A: 42.4 (20.0); 45.1 (21.0) TMT-B: 98.9 (43.0); 99.8 (39.5)	There were significant differences in TMT-A and TMT-B between groups after 4 weeks of intervention (*p* = 0.009, *p* = 0.006, respectively)	Yes
	Executive functions: The digit span test (DST)			
Park (2019) [18]	Mean DST at: baseline; 24th week follow up	Gr1: 2.7 (0.2); 2.4 (0.2) Gr2: 2.6 (0.3); 2.9 (0.2)	There were significant differences in DST between groups after 24 weeks of intervention (*p* = 0.02)	Yes
Donnezan et al. (2018) [19]	DSF and DSB at: baseline; 6 months follow up.	Gr1: 5.38 (1.14); 5.94 (0.87) Gr2: 5.18 (0.91); 6.18 (1.11) Gr3: 5.48 (0.88); 6.15 (1.06) Gr4: 5.21 (1.12); 5.36 (0.84)	DSF and DSB tests showed significant differences after PT, CT, and PCT interventions.	Yes
	Executive functions: Stroop test			
Liao et al. (2019) [21]	Stroop color and word test (number; time) at: baseline; 12 week follow up	Gr1: Number: 15.05 (6.59); 19.44 (9.05); Time: 126.83 (41.03); 100.66 (33.93) Gr2: Number: 126.83 (41.03); 100.66 (33.93); Time: 119.87 (54.35); 100.18 (41.89)	VR and PCT groups exhibited significant improvements in Stroop test scores, but there was no significant difference between the groups.	Yes within group No deferent group
Anderson-Hanley et al. (2018) [22]	Stroop A/C at: baseline; 6th month follow up	Gr1: 0.40 (0.12); 0.48 (0.11) Gr2: 0.40 (0.14); 0.47 (0.15)	The results showed that StroopA/C improved significantly in both the exer-tour (*p* = 0.049) and exer-score conditions (*p* = 0.001) and between groups (*p* = 0.002)	Yes within group No deferent group
Donnezan et al. (2018) [19]	Stroop test: task switching (number) at: baseline; 6 month follow up.	Gr1: 28.89 (6.45); 30.94 (6.24) Gr2: 27.19 (8.82); 25.5 (8.37) Gr3: 26.52 (6.8); 29.05 (7.19) Gr4: 24.71 (10.16); 26.42 (6.53)	The finding indicated an improvement in Stroop test scores after combined PCT, but the difference was not statistically significant between groups (*p* = 0.06)	Yes within group No deferent group

IG, intervention group; CG, control group; Gr, group; s, second; MMSE, Mini Mental State Examination; MoCa, Montreal Cognitive Assessment; Korean version of the KMMSE; TUG, timed-up-and-go; DST, digit span test; TMT, trail-making test; TMT-B; ADAS-Cog, modified Alzheimer’s Disease Assessment Scale Cognitive Subscale (ADAS-Cog); SCWT, Stroop color and word test; StroopA/C, ratio of the executive function component of the task; DSF, digit span forward test; DSB, digit span backward test; PT, physical training; CT, cognitive training; PCT, physical and cognitive training; VR, virtual reality.

## Data Availability

All data generated during this study are included in this published article (Table 1 and Table 3).

## Supplementary Materials

The following supporting information can be downloaded at: https://www.mdpi.com/article/10.3390/healthcare10050862/s1, Table S1: Reasons for studies being excluded from the final analysis [31,32,33,34,35,36,37,38,39,40,41,42,43,44,45,46,47,48,49,50,51,52,53,54,55,56,57,58,59,60,61,62,63,64,65,66,67,68].
