# Supplementary material for: The Effects of Combined Physical and Cognitive Interventions on Direct and Indirect Fall Outcomes for the Elderly with Mild Cognitive Impairment: A Systematic Review
Source: Healthcare (Basel). 2022 May 6;10(5):862. doi: 10.3390/healthcare10050862 (PMC9140643; doi:10.3390/healthcare10050862)
Supplement: Supplementary file 1 [file healthcare-10-00862-s001.zip › healthcare-1662149-supplementary.pdf]

Supplementary Material

Table S1. Reasons for studies being excluded from the final analysis.

**Method (*n* = 4)**	**Intervention (*n* = 7)**	**Outcomes (*n* = 16)**
Smith and Argentina [31]	Suzuki, Shimada [32]	Klados, Styliadis [33]
Mirza and Yaqoob [34]	Hong, Kim [35]	Kim and Kim [36]
Anderson-Hanley, Stark [37]	Kovács, Sztruhár Jónásné [38]	Boa Sorte Silva, Gill [39]
Konstantinidisa, Billisa [40]	Thaiyanto, Sittichoke [41]	Park [42]
Unable to obtain (*n* = 2)	Whitney, Jackson [43]	Mrakic-Sposta, Di Santo [44]
Kato, Kanemaru [45]	Sugano, Yokogawa [46]	Jeong, Jung [47]
Sacco, Caillaud [48]	de Oliveira Silva, Ferreira [49]	Maffei, Picano [50]
Population (*n* = 6)	Other issues (*n* = 3)	Gonzalez-Palau, Franco [51]
Klotzbier and Schott [52]	Hagovska and Olekszyova [53]	Wall, Stark [54]
Barcelos, Shah [55]	Hagovska and Nagyova [56]	de Boer, Echlin [57]
Shatil [58]	Hagovská, Takáč [59]	Nishiguchi, Yamada [60]
Desjardins-Crepeau, Berryman [61]		Damirchi, Hosseini [62]
Adcock, Thalmann [63]		Lisanne, Best [64]
Titheridge [65]		Makizako, Doi [66]
		Fiatarone Singh, Gates [67]
		Kobe, Witte [68]
